# The Role of Microorganisms and Carbon-to-Nitrogen Ratios for Microbial Protein Production from Bioethanol

**DOI:** 10.1128/aem.01188-22

**Published:** 2022-10-26

**Authors:** L. Van Peteghem, M. Sakarika, S. Matassa, K. Rabaey

**Affiliations:** a Center for Microbial Ecology and Technology (CMET), Faculty of Bioscience Engineering, Ghent University, Gent, Belgium; b Center for Advanced Process Technology for Urban Resource Recovery (CAPTURE), Gent, Belgium; c Department of Civil, Architectural, and Environmental Engineering, University of Naples Federico II, Naples, Italy; Washington University in St. Louis

**Keywords:** microbial food, bioethanol, microbial protein, C/N ratio, alternative protein

## Abstract

With industrial agriculture increasingly challenging our ecological limits, alternative food production routes such as microbial protein (MP) production are receiving renewed interest. Among the multiple substrates so far evaluated for MP production, renewable bioethanol (EtOH) is still underexplored. Therefore, the present study investigated the cultivation of five microorganisms (2 bacteria, 3 yeasts) under carbon (C), nitrogen (N), and dual C-N-limiting conditions (molar C/N ratios of 5, 60, and 20, respectively) to evaluate the production (specific growth rate, protein and biomass yield, production cost) as well as the nutritional characteristics (protein and carbohydrate content, amino acid [AA] profile) of MP production from bioethanol. Under C-limiting conditions, all the selected microorganisms showed a favorable AA profile for human nutrition (average AA score of 1.5 or higher), with a negative correlation between protein content and growth rate. Maximal biomass yields were achieved under conditions where no extracellular acetate was produced. Cyberlindnera saturnus and Wickerhamomyces anomalus displayed remarkably high biomass yields (0.40 to 0.82 g cell dry weight [CDW]/g EtOH_consumed_), which was reflected in the lowest estimated biomass production costs when cultivated with a C/N ratio of 20. Finally, when the production cost was evaluated on a protein basis, Corynebacterium glutamicum grown under C-limiting conditions showed the most promising economic outlook.

**IMPORTANCE** The global protein demand is rapidly increasing at rates that cannot be sustained, with projections showing 78% increased global protein needs by 2050 (361 compared to 202 million ton_protein_/year in 2017). In the absence of dedicated mitigation strategies, the environmental effects of our current food production system (relying on agriculture) are expected to surpass the planetary boundaries—the safe operating space for humanity—by 2050. Here, we illustrate the potential of bioethanol—renewable ethanol produced from side streams—as a main resource for the production of microbial protein, a radically different food production strategy in comparison to traditional agriculture, with the potential to be more sustainable. This study unravels the kinetic, productive, and nutritional potential for microbial protein production from bioethanol using the bacteria Methylorubrum extorquens and Corynebacterium glutamicum and the yeasts *Wickerhamomyces anomalus,* Cyberlindnera saturnus, and Metschnikowia pulcherrima, setting the scene for microbial protein production from renewable ethanol.

## INTRODUCTION

Global meat consumption reached about 327 million tons in 2018 (1), representing a 58% increase over the last 20 years (2). Only 54% of this increase can be attributed to population growth, while the remainder is a direct consequence of the growing meat consumption per capita (2). By 2030, meat consumption is expected to increase by another 14% compared to the average meat consumption in 2018 to 2020 (1), exacerbating deforestation and biodiversity loss (3, 4), eutrophication of terrestrial and aquatic systems (5–7), depletion of freshwater resources (8, 9), and climate change (10). These challenges are motivating the search for alternative protein sources such as plant-based protein (e.g., soybean), insects, cultured meat, and microbial protein (MP), i.e., the protein-rich microbial biomass from bacteria, microalgae, yeasts, or filamentous fungi. The development of these products aims at securing the future protein supply while reducing meat production and mitigating the environmental impact of our food system.

Over the past decades, various feedstocks and by-products have been considered as substrates for MP production. Current examples of industrially produced microbial foods include Quorn (Marlow Foods, United Kingdom) from glucose, Fy (Nature’s Fynd, USA) from starch and sugars, and Vegemite (Bega Cheese Limited, Australia), a by-product from beer brewing. Even though the majority of MP production for food purposes relies on carbohydrates, other organic feedstocks largely employed in food manufacturing could become an attractive option for MP synthesis. A recent technical assessment showed that MP production from bioethanol derived from CO_2_ and H_2_ can potentially accomplish a negative carbon footprint by 2030 (11). From an integrated biorefinery perspective, bioethanol fermentation from industrial waste streams and its subsequent upgrade into MP could provide several advantages, such as the avoidance of the energy-intensive concentration step needed to remove water and other organic acids (e.g., acetate), as MP can be produced directly from diluted ethanol solutions (6% for gas fermentation [12] and 8% for biowaste fermentation [11, 13]). In addition, being completely miscible in water and having a low oxygen demand and heat release, the aerobic oxidation of ethanol can reduce the energy consumption of MP fermentation compared to that of other hydrocarbon substrates (14). Therefore, based on its environmental sustainability, high availability, and technical advantages, bioethanol stands as a promising substrate for MP production, deserving further investigation.

Various bacteria and yeast have received attention in relation to MP from ethanol (15). Although a multitude of organisms have been reported to be able to utilize ethanol as an energy and carbon source (14), only a limited number of species have been examined and/or industrially utilized to produce MP (e.g., Candida utilis, Hansenula anomola, Candida ethanothermophilum, and Acinetobacter calcoaceticus). Moreover, most of the research on MP from ethanol dates back to the 1970s, and the most recent scientific study investigating the use of ethanol as a substrate for MP is from 1996 (16). Since then, more and more ethanol-utilizing microorganisms, including both yeasts and bacteria, have reached the “generally recognized as safe” (GRAS) status, and yet a clear evaluation of their potential for MP production from ethanol is lacking.

To our best knowledge, the experimental investigation of specific growth rate and biomass and protein yields of ethanol-utilizing microorganisms employing different metabolic pathways (e.g., glyoxylate cycle, ethylmalonyl-CoA pathway) has not yet been carried out. As such, the present study aimed to compare the MP production potential of five selected microbial species (i.e., yeasts and bacteria that cover both carbon metabolisms). The microorganisms were selected based on their ability to assimilate ethanol and their association with food and beverage production. More specifically, Methylorubrum extorquens is currently used by KnipBio to produce MP as an aquafeed ingredient (17). Wickerhamomyces anomalus has been indicated as one of the most promising organisms for MP production from ethanol (18). Corynebacterium glutamicum is widely employed for industrial amino acid (AA) production (19). Finally, both Cyberlindnera saturnus and Metschnikowia pulcherrima have been used to modulate the sensory profile and ethanol content of wine (20, 21). The selected species were evaluated on their production (i.e., specific growth rate, protein and biomass yield, and production cost) and nutritional characteristics (i.e., protein and carbohydrate content, balanced AA profile for human nutrition). Even though industrial MP production would be most likely performed under C-limiting conditions to minimize substrate losses, the optimal use of both ethanol and nitrogen could increase the overall economic and environmental sustainability of the process. Accordingly, the impact of ethanol-limiting (C/N 5 [mol/mol]) and nitrogen-limiting (C/N 60 [mol/mol]) conditions, as well as the anticipated dual C and N limitation (C/N 20 [mol/mol]) on the MP production process, was investigated.

## RESULTS

### Biomass and protein yields.

**FIG 1:**
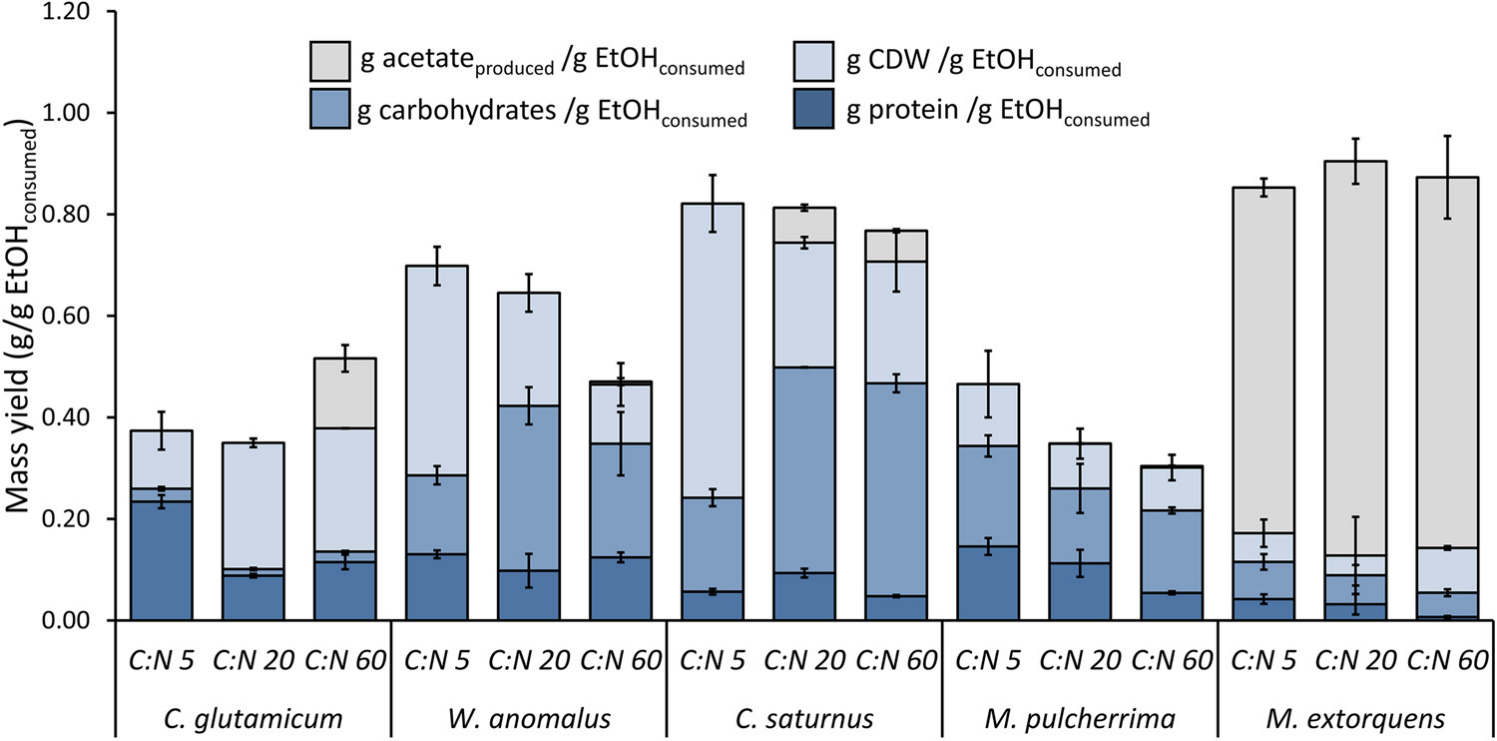
The yield in terms of protein, carbohydrates, and biomass (sum of protein, carbohydrates, and other nonmeasured components such as lipids and ash) based on ethanol consumption. The amount of excreted acetate, still present in the supernatant in the stationary phase, is also expressed per gram of ethanol consumed. Average values are presented, and error bars represent the ± standard deviation (*n* = 3).

Of all the tested microorganisms, *W. anomalus* (0.64 and 0.70 g cell dry weight [CDW]/g EtOH_consumed_, molar C/N ratio of 20 and 5, respectively) and *C. saturnus* (0.71 to 0.82 g CDW/g EtOH_consumed_, all tested initial C/N ratios) had the highest biomass yield (Fig. 1). M. extorquens (0.14 to 0.23 g CDW/g EtOH_consumed_) had the lowest biomass yield. In some cases, a portion of the initial ethanol was converted into extracellular acetate. For M. extorquens, this fraction amounted to 52 to 60 mol% of the consumed ethanol; it was 11 mol% for C. glutamicum and 5 mol% for *C. saturnus*.

Regardless of the initial C/N ratio, both yeasts presented a low protein yield (*W. anomalus*: 0.10 to 0.13 g protein/g EtOH_consumed_; *C. saturnus*: 0.048 to 0.093 g protein/g EtOH_consumed_). In contrast, C. glutamicum and *M. pulcherrima* reached the highest protein yield (C. glutamicum: 0.088 to 0.23 g protein/g EtOH_consumed_; *M. pulcherrima*: 0.054 to 0.15 g protein/g EtOH_consumed_). The lowest protein yield was achieved with M. extorquens (0.0071 to 0.042 g protein/g EtOH_consumed_).

### Growth and protein kinetics.

**FIG 2:**
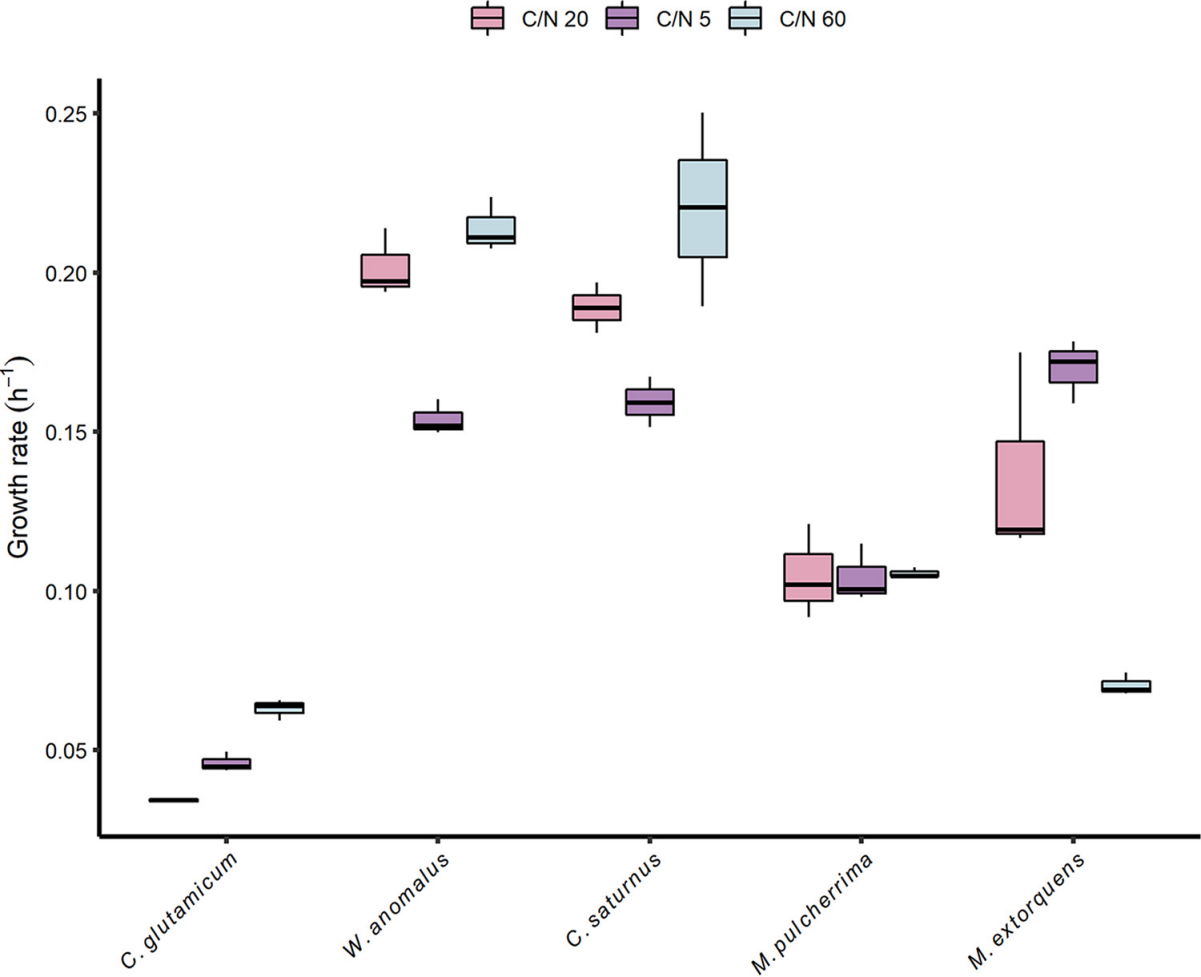
Specific observed growth rate of the different microorganisms grown in batch mode on ethanol with various initial C/N molar ratios. The median of the biological replicates is shown as a horizontal black bar inside the boxplot (*n* = 3).

The specific growth rates of *M. pulcherrima* (0.11 ± 0.01 h^−1^) and C. glutamicum (0.048 ± 0.013 h^−1^) were the lowest among the tested species and showed relatively small variation over the different C/N ratios (Fig. 2). The opposite was observed for *W. anomalus*, *C. saturnus*, and M. extorquens. W. anomalus and C. saturnus reached the highest growth rates and showed a positive trend with decreasing nitrogen concentration. For M. extorquens, the specific growth rate decreased with an increasing C/N ratio.

**FIG 3:**
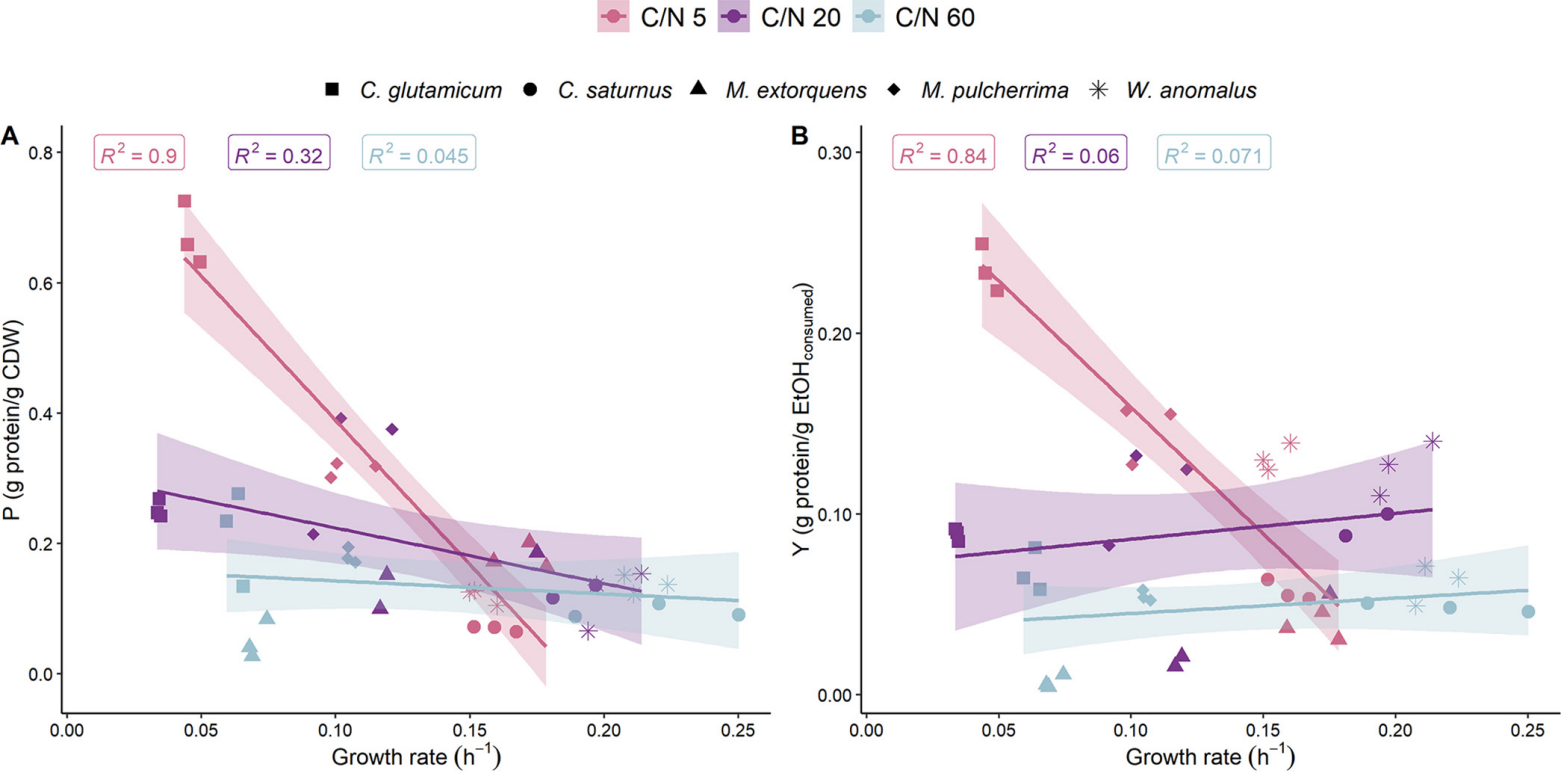
(A and B) Effect of the specific observed growth rate on protein content (A) and protein yield (B) of all tested microbial species indicated by different shapes. The results of all replicates (*n* = 3) are plotted under the different tested C/N ratios. The straight line represents the fitted linear regression model of which the R^2^ value is indicated in the graph. The shaded regions show the 95% confidence intervals. The Pearson correlation coefficient (*r*), Spearman’s rank correlation coefficient (*ρ*) and *P* value can be found in Table SA5.

The protein content of the biomass decreased with increasing specific growth rate regardless of the tested microbial species for the initial C/N ratios of 5 and 20 (Fig. 3A; see File SA5 in the supplemental material). This negative correlation was stronger for the C/N ratio of 5 (R^2^ = 0.899; *P* = 4.8E-08) in comparison to C/N ratios of 20 (R^2^ = 0.36; *P* = 0.036). In the case of an initial C/N ratio of 60, no correlation between protein content and specific growth rate was found. The correlation between protein yield and growth rate was negative for the initial C/N ratio of 5, while no correlation could be found for C/N ratios of 20 and 60 (Fig. 3B).

### Nutritional quality of the biomass.

**FIG 4:**
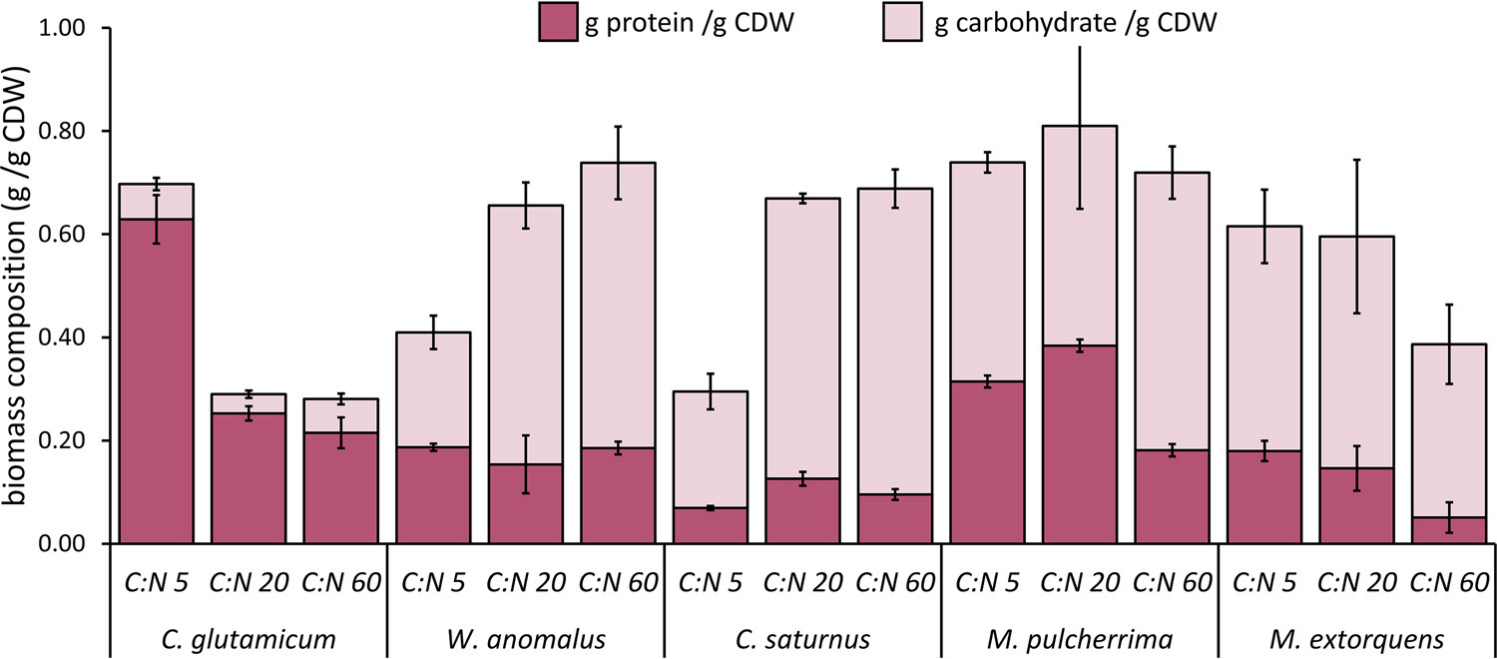
Overview of the protein and carbohydrate content for each tested species grown on ethanol with varying initial C:N molar ratios. The bars represent average values ± standard deviation (*n* = 3) depicted as error bars.

Among the tested microorganisms, C. glutamicum had the highest protein content, 63 ± 4.7% under C-limiting conditions (Fig. 4). Similar to the protein yields, *M. pulcherrima* had the second-highest protein content when grown under an initial C/N ratio of 5 (31 ± 1.2%) and 20 (33 ± 9.8%). The protein content of *W. anomalus* and *C. saturnus* ranged between 6.9 and 19% under the different conditions. In contrast to the protein content, C. glutamicum had the lowest carbohydrate content (3.7 to 6.8%), whereas the carbohydrate content varied between 22 and 59% for the examined yeasts. M. extorquens had a similar protein content for the initial C/N ratios of 5 and 20 (18 and 15%, respectively), while under N-limiting conditions, it had the lowest protein content of all tested species (5.1%). The carbohydrate content of M. extorquens biomass was relatively constant for the initial C/N ratios of 5 and 20 (44 to 45%), while at the initial C/N ratio of 60, carbohydrates amounted up to 34% of the total biomass.

**FIG 5:**
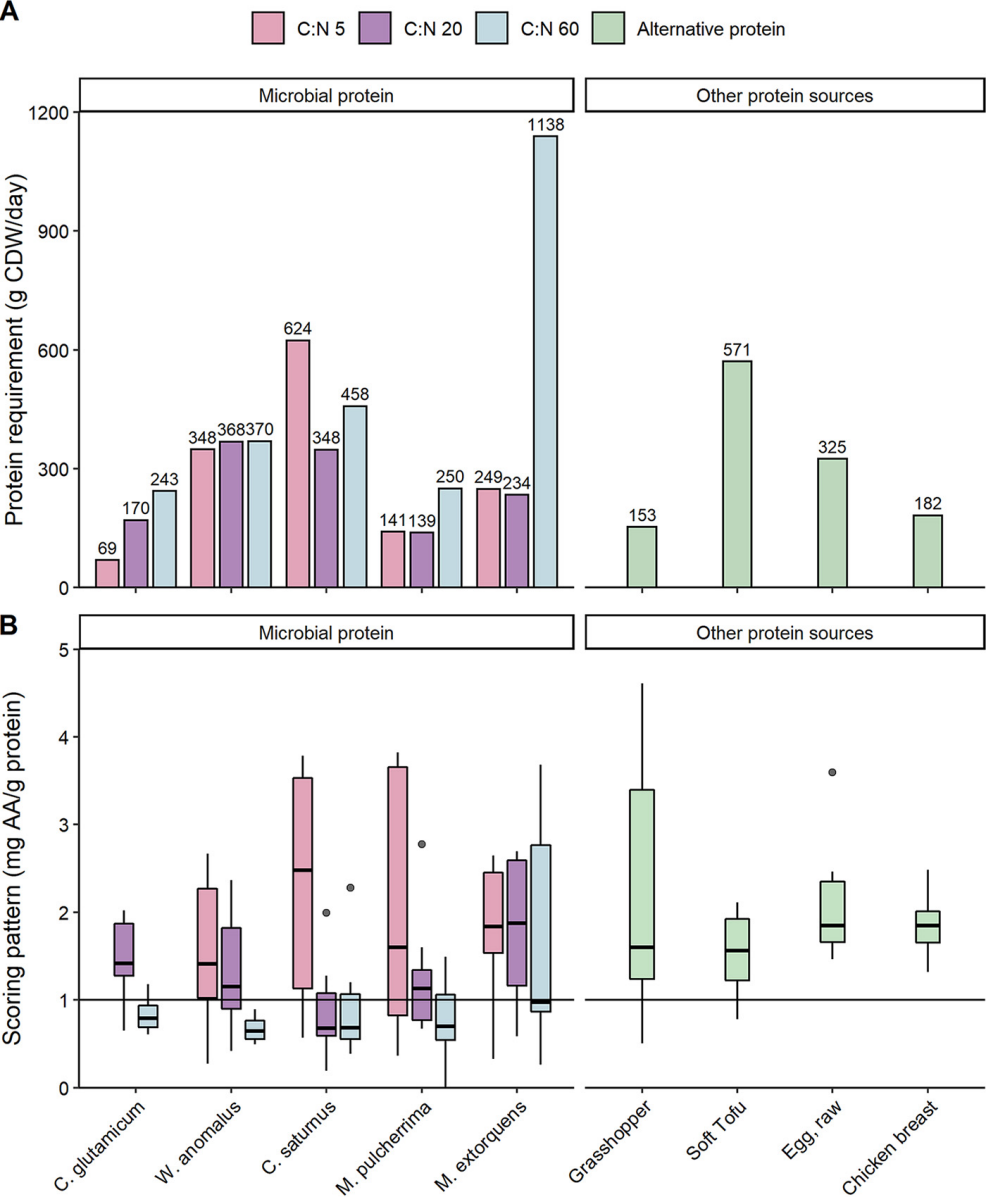
(A) Quantity of the average microbial biomass (*n* = 3) (considering a final moisture content of 5%) needed to cover the daily total protein requirements of an average adult weighing 62 kg (58) as defined by the World Health Organization and United Nations University (22). (B) Scoring pattern for the essential AA content to match human nutrition from microbial protein and other protein sources (a perfect match is represented by a score of 1) (22). Outliers are shown with black dots outside the boxplot, and the median is shown as a horizontal black bar inside the plots. C. glutamicum with an initial C/N ratio of 5 was not included due to poor data quality. The data used to calculate the daily total protein requirements and scoring patterns of alternative protein were obtained from (60) and the U.S. Department of Agriculture (59) (Table SA6).

To meet the daily recommended protein intake, an average-weight adult should consume 69 g of biomass from C. glutamicum grown under C-limiting conditions (i.e., C/N ratio of 5) (Fig. 5A). In the case of *M. pulcherrima*, one would need to consume about double the amount of biomass, i.e., ≈140 g. Finally, relatively high required amounts of *C. saturnus* (C/N 5) and M. extorquens (C/N 60) (620 and 1,100 g, respectively) would be required for the same purpose.

Along with recommendations on the total intake of protein, the World Health Organization and the United Nations University (22) proposed reporting the suitability of protein sources for human nutrition as a scoring pattern based on the AA profile (Data Set S1). The average scores for microorganisms grown under C-limiting conditions were generally higher (1.53 to 2.33) than those under the other conditions (0.69 to 2.07). Overall, the variance for organisms grown with an initial C/N ratio of 20 and 60 was relatively low (0.021 to 0.38, excluding M. extorquens), while for the examined yeasts at C/N 5 and M. extorquens (under all conditions), the variance was substantially higher, ranging from 0.70 to 2.2 (Fig. 5B).

### Preliminary techno-economic evaluation.

**FIG 6:**
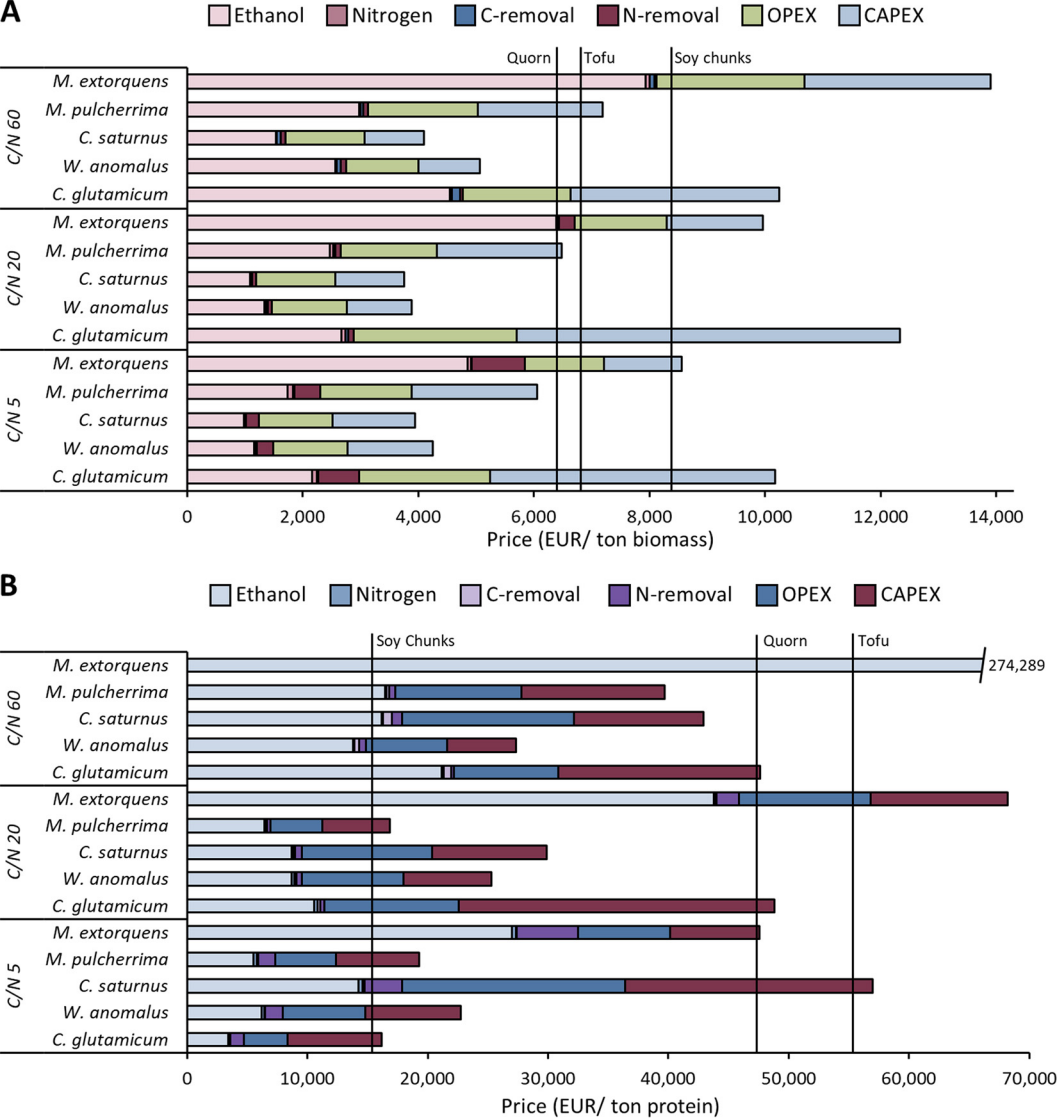
Average cost estimation of MP produced in a continuous process with a 150-m^3^ bioreactor based on the experimentally obtained yields and rates. (A) Production cost on a biomass basis. (B) Cost estimation on a protein basis. The estimated cost for the MP products presented here is the production price of a food-grade powder (4% water content). The prices for soy chunks, Quorn, and tofu are the selling prices of the finished products (foodie, 2020a, 2020b; Tesco, 2020) (61).

MP production from *C. saturnus* and *W. anomalus* grown at a balanced initial C/N ratio of 20 shows the best economic outlook for biomass production among all tested conditions (Fig. 6A), with an estimated production cost of 3,762 and 3,888 EUR/ton biomass, respectively. *M. pulcherrima* and M. extorquens should instead be grown with an initial C/N ratio of 5 to reach production costs of 6,060 and 8,554 EUR/ton biomass, respectively, while a C/N ratio of 60 for C. glutamicum would allow its production at 10,177 EUR/ton biomass. When protein, and not biomass, is taken as a reference for the cost comparison, the preferable C/N ratios and microbial species change. C. glutamicum at a C/N ratio of 5 and *M. pulcherrima* at a C/N ratio of 20 represent the cheapest protein production routes (16,184 and 16,878 EUR/ton protein, respectively) (Fig. 6B).

The cost of ethanol as a substrate represents the largest cost item, accounting for about 20 to 63% of the total MP production cost. This cost largely depends on the experimentally obtained yields. The capital expenses (CAPEX) are mainly determined by the specific growth rate and represent 16 to 54% of the total MP production cost. The operational expenses (OPEX) are dominated by the energy requirement (6.3 to 24% of the total cost) and by personnel (4.7 to 16% of the total cost) (Data Set S1). The latter is calculated as a fixed cost per unit volume of the bioreactor and, consequently, the variability in OPEX costs can be attributed to the change in volumetric productivity for each process condition considered. Under C-limiting conditions, N removal represents between 5.7 and 11% of the total cost, while for the other tested conditions, this is between 0.24 and 2.9%. The smallest share of costs is ascribed to C removal (0.072 to 1.5%).

## DISCUSSION

### C. glutamicum and *M. pulcherrima* demonstrate the highest potential for microbial protein production based on yields and biomass quality.

The biomass yields of *C. saturnus* and *W. anomalus* under C-limiting conditions were among the highest reported in the literature thus far (14, 16). However, the largest share of the consumed substrate was not used for protein production, as indicated by their low protein yields (Fig. 1). The high protein yield of C. glutamicum and *M. pulcherrima* makes these two microorganisms more interesting from a protein production perspective (Fig. 1). Mor and Fiechter (23) reported protein yields for Saccharomyces cerevisiae grown on ethanol of between 0.17 and 0.27 g protein/g EtOH_consumed_ for a continuous process with various dilution rates (0.02 to 0.12 h^−1^). Abbott et al. (24) reached protein yields of 0.44 to 0.46 g protein/g EtOH_consumed_ using Acinetobacter calcoaceticus, a species used by a large-scale project from Exxon-Mobile and Nestlé to produce MP from ethanol for human consumption, in a continuous setup (15). These values are up to two times higher than the highest yields reached in this study. An important side note, however, is that both studies determined the protein yields based on the total Kjeldahl nitrogen in the biomass (25). This method measures the total organic nitrogen in the biomass, also comprising nucleic acids and amines, which can lead to the overestimation of the protein content (26), in contrast to this work, where the protein concentration was determined based on the bicinchoninic acid (BCA) method. This method measures the peptide bonds and specific AA (cysteine, cystine, tryptophan, and tyrosine); i.e., nucleic acid and amines are not measured. Furthermore, this method is less affected by protein compositional differences, whereas the Kjeldahl method assumes protein with 16% (wt/wt) N for all samples (26). In addition, even though Exxon-Mobile and Nestlé showed a particular interest in A. calcoaceticus, its potential as a food product today could be hampered by the fact that bacteria belonging to the Acinetobacter genus show multidrug-resistant features and are classified as biosafety level 2 (27, 28).

Aside from the protein and biomass yields, the protein content, which is one of the main factors affecting the quality of the final MP product, differed substantially among the selected species and conditions. The protein content of C. glutamicum, under C-limiting conditions (63 ± 4.7%), the highest from this study, is similar to that of A. calcoaceticus (67 ± 0.75%) (24). All yeasts produced in this study showed lower protein contents than S. cerevisiae (42 to 58%) grown on ethanol in a continuous process (23). The analysis of the results from a nutritional perspective indicates that the amount of biomass from *M. pulcherrima* (grown on medium with an initial C/N ratio of 5 or 20) and C. glutamicum (grown on medium with an initial C/N ratio of 5) that would be needed to meet the recommended protein intake is below that of other alternative protein sources (i.e., insects and soybeans) (Fig. 5). In addition, an average-weight adult would need to consume 2.6 times less biomass from C. glutamicum (grown on medium with a C/N ratio of 5) than chicken (raw broiler breast) (Fig. 2B). The microbial biomass produced in this study had a less evenly distributed AA scoring pattern due to the lack of specific essential AAs. Specifically, lysine (0.26 to 1.5), methionine (0.27 to 1.8), and leucine (0.46 to 1.8) did not meet the standards for human nutrition in the majority of the MP samples, evidently assuming that the MP is used as single protein source. For the alternative protein sources reported here, the limiting amino acid was methionine for tofu, while grasshoppers contained low amounts of cysteine and isoleucine. None of the tested microorganisms appeared to have a score higher than 1 for all amino acids. However, C. glutamicum (C/N, 20) and M. extorquens (C/N, 20) reported an AA score below 1 only for methionine (AA score, 0.65) and lysine (AA score, 0.59), respectively. Considering all the production as well as the nutritional characteristics investigated in this study, both C. glutamicum and *M. pulcherrima* seem to be promising candidates for MP production from ethanol. Interestingly, the latter has not been traditionally considered for ethanol utilization or protein or AA production.

### Biomass components different from protein can boost the product quality of microbial protein.

Even though protein is considered the most valuable product contained in the microbial biomass when it comes to food production, other macro elements such as carbohydrates and lipids can also play a role in human nutrition. It is known that by limiting nitrogen, protein synthesis can be restricted, given that nitrogen is a crucial element in AA synthesis (29). This decreasing trend in protein content is confirmed by the results in this study (Fig. 4). Unlike the yeast biomass, the biomass of C. glutamicum did not contain more carbohydrates at an initial ratio of C/N 5 compared to C/N 60; the content remained below 6.8% for the three conditions. It has been reported that C. glutamicum produces polyhydroxyalkanoate (PHA) when grown on acetate under N-limiting conditions (30). Given that acetate is an intermediate product during ethanol assimilation, it is likely that PHA is also produced when grown on ethanol.

The Institute of Medicine (Washington, DC, USA) recommends a daily intake of 130 g of carbohydrates for adults (31). Depending on the type (e.g., sugars, oligosaccharides, or polysaccharides) and digestibility of carbohydrates from the yeast biomass, the yeast MP product (considering a 5% water content) from this study could be a nearly complete human food source in terms of energy (carbohydrate content of 22 to 59%) and protein content (6.6 to 36%) in comparison to meat, which typically contains 0 to 5.2% (wt/wt) carbohydrates and has a protein content of 9.7 to 36% (32).

Taking into account multiple nutritional aspects of the biomass shows that the nutritional properties of MP biomass are not only limited to the protein fraction. As a result, from a food production perspective, species such as *W. anomalus* and *C. saturnus* grown with initial C/N ratios of 20 and 60 and having a lower protein content (10 to 19%), can still be interesting for food production given their high biomass yields (Fig. 1) and carbohydrate content (Fig. 2).

### Understanding ethanol metabolism to improve production processes.

The carbon metabolism used for ethanol or acetate assimilation differs substantially from carbohydrate and C_1_ molecule (e.g., CH_4_, CH_3_OH) assimilation, which are currently among the most explored substrates for MP production (33). Both ethanol and acetate enter the central carbon metabolism via the precursor metabolite acetyl-CoA, which can be converted via the glyoxylate cycle (34) or the ethylmalonyl-CoA pathway (35) to cell carbon constituents (e.g., protein and carbohydrates) (36). The latter is considered more thermodynamically efficient than the former for growth on acetate (38 to 55% for the glyoxylate cycle on acetate versus 60 to 65% for the ethylmalonyl-CoA pathway on acetate) (37). Surprisingly, M. extorquens, the only species tested in the present study that utilizes the ethymalonyl-CoA pathway to metabolize ethanol (38), had the lowest biomass and protein yield. These lower yields could be explained by the fact that 52 to 60 mol% of the consumed ethanol was converted into extracellular acetate instead of protein or other biomass, as occurred for all other tested microorganisms (Fig. 1). This acetate accumulation in the medium resulted in a quick drop in the pH from 6.8 to 5.3 and inhibited growth. Only about 1 g EtOH/L was consumed in all tested conditions. All other species also showed extracellular acetate production, but solely under N-limiting conditions and to a much lower extent than M. extorquens. It is assumed that under these conditions, ethanol was still oxidized to sustain its catabolic needs, but further assimilation of acetate was hindered by N limitation.

Ristroph et al. (39) determined that the ethanol concentration should be kept below 4 g EtOH/L in a fed-batch operation with Candida utilis to prevent extracellular acetate production and subsequent lower biomass yield. For all experimental conditions, it was therefore decided to work at an initial concentration of 4 g EtOH/L to verify whether the selected species would produce extracellular acetate at such concentrations as well as to avoid ethanol toxicity (e.g., ethanol toxicity for A. calcoaceticus is 10 g EtOH/L) (40). Based on the above, it could be concluded that for M. extorquens, which utilizes the ethylmalonyl pathway, a concentration of 4 g EtOH/L is too high to prevent acetate accumulation under C-limiting conditions. In contrast, this concentration was sufficiently low for *C. saturnus*, C. glutamicum, and *W. anomalus* to prevent extracellular acetate production under C-limiting conditions. However, for *M. pulcherrima*, acetate accumulation did occur during the batch growth, up to 8.0 to 14% of the total consumed ethanol in all conditions (Fig. SA1). This acetate was eventually consumed again when the ethanol concentration decreased. Watteeuw et al. (41) indicated that the lower biomass yield from *C. utilis* on acetate could be attributed to a higher maintenance coefficient than that of *C. utilis* grown on ethanol. In accordance, *M. pulcherrima* had the lowest biomass yield in comparison to the other examined yeasts. The results obtained here indicate that one of the possible strategies to maximize yeast biomass yields on ethanol could be the minimization of extracellular acetate production. For *M. pulcherrima* this could be achieved by a coculture, which might result in higher process stability. However, if a maximal protein yield is desired, the impact of this acetate production on the final protein content should be further examined, given that *M. pulcherrima* had the highest protein yield from all tested yeasts.

The biomass productivity of an aerobic heterotrophic MP production process in a bioreactor depends on the maximal specific growth rate, oxygen transfer rate (OTR), and heat removal. Both the OTR and heat removal are determined by the equipment employed, while the specific growth rate is linked to the microorganism and nutrient and carbon availability (42, 43). Apart from the volumetric productivity, the growth rate could also impact the protein content of the biomass. This aspect has been discussed multiple times in the framework of MP production from ethanol, giving rise to contradicting findings. A. calcoaceticus, for instance, was reported to have an increased protein content with increasing dilution rate and, hence, increasing growth rates under C-limiting conditions (15). On the other hand, the same organism has also been reported to have a decreased protein content at higher growth rates under similar C-limiting conditions (44). Du Preez et al. (44) and Yech (16) indicated a positive correlation between protein yield/content and growth rate for, respectively, A. calcoaceticus and *Rhodotorula* sp. strain Y-38 grown on ethanol. The experimental data gathered in this study indicate a negative correlation between protein content/yield and specific growth rate over the different tested species at C-limiting conditions (Fig. 3A). This indicates that selecting a microorganism and operational conditions by targeting a high specific growth rate might undermine the protein production potential. This also suggests that when biomass was produced at a high rate, the organisms invested more energy into biomass components such as nucleic acids, lipids, or carbohydrates rather than protein. Therefore, if the final protein content is of secondary importance to the total biomass productivity, choosing a microorganism with a high growth rate could be a preferable option. More specifically, high growth rates will also lead to higher volumetric productivities, thereby lowering the overall MP production costs.

### Industrial MP production from bioethanol: preliminary techno-economic considerations.

Abbott et al. (24, 45) and Laskin (15) indicated that MP production from A. calcoaceticus should be operated under C-limiting conditions and at high growth rates to maximize biomass and/or protein yield. However, optimal uptake of other nutrients, such as nitrogen, can also play a key role in establishing a cost-efficient process. The cost to remove organic nitrogen from wastewater via classic nitrification/denitrification amounts to about 3 EUR/kg N (46). The tests performed with an excess of nitrogen (C/N ratio of 5) resulted, in the present study, in overall higher biomass and protein yields than in those performed with a C/N ratio of 20, which is closer to the requirements of balanced microbial growth. In spite of this, the estimated production costs for *C. saturnus* and *W. anomalus* with an initial C/N ratio of 20 is, respectively, 1.05 and 1.10 times lower, mainly due to smaller CAPEX, related to the higher specific growth rate, and a reduced cost for N removal (5.0 and 7.9 times less for *C. saturnus* and *W. anomalus*, respectively). It is important to highlight that during the downstream processing, a fraction of the separated water can be recycled, therefore enabling the recovery of key nutrients such as nitrogen. However, the maximum amount of water that can be recycled depends on the concentration of the incoming ethanol stream. The present study aimed to illustrate the use of bioethanol solutions, which can be produced at concentrations between 6% (gas fermentation) (12) and 8% (biowaste fermentation) (13, 47) without distillation. As a result, only a limited amount of the water can be recycled to avoid further dilution of the substrate and consequent reduction of the maximum achievable biomass concentration in the reactor. In summary, due to the use of bioethanol solutions, higher volumes of wastewater are produced, less N is recycled, and the costs to remove residual N can have a significant impact on the economics of the whole process (0.24 to 11% of the total cost). In addition, a lower biomass concentration inside the reactor also results in higher energy requirements; i.e., more water needs to be removed downstream, a larger reactor effluent volume must be heat-treated and aeration, agitation, and cooling requirements per unit of biomass are higher given the lower productivity in the bioreactor.

Antoniak et al. (48) indicated that food labels implying a high protein content could have a positive impact on consumers’ perception. In other words, a product’s protein content is valued by the consumer. In light of this, the economic comparison based on the cost of protein production rather than biomass showed that the species C. glutamicum and *M. pulcherrima* could yield the most cost-efficient process (Fig. 6B), a contradictory conclusion in relation to the cost expressed per ton of biomass. The final choice will thus depend on how the MP product will be positioned in the food market. Should the product be marketed as a food ingredient, functioning as the primary protein source, the relevant production price to be considered should be the one calculated per unit of protein. If MP is instead introduced as a food product on its own, as it is done for Quorn (protein content, 11 to 15%), the pricing per ton of biomass would be more relevant.

The main factors contributing to the final costs are the expenses related to purchased bioethanol and the CAPEX (Data Set S1). In other words, the choice of bioethanol as a substrate, the yield on ethanol, and the specific growth rate are the three key parameters determining the overall costs. In comparison to sugars (0.30 ± 0.056 EUR/kg sugar), the ethanol price has been substantially higher over the past decade (0.55 ± 0.14 EUR/kg EtOH) (49). This could hamper the economic competitiveness of using bioethanol as a substrate for MP production over the conventionally used sugars. However, in a context where land use and land use change are becoming more and more important in view of sustainable food production (50), the potential of ethanol production directly from CO_2_ and H_2_ (51) could enable a process that is decoupled from traditional agricultural practices, thereby reducing land occupation (52, 53).

**Conclusion.** Of all the tested organisms, C. glutamicum and *M. pulcherrima* showed the highest potential toward protein-rich biomass production from ethanol, considering protein content, AA score, and price. Overall, the microorganisms grown on ethanol under carbon-limiting conditions showed a favorable AA profile for human nutrition, with an average AA score of 1.5 or higher. A negative correlation between protein content and growth rate was observed over the different tested species at C-limiting conditions. Maximal biomass yields could be achieved under conditions where no extracellular acetate is produced. Starting the MP production process from bioethanol solutions (6 to 8% (w/w)), higher volumes of wastewater are produced, less N is recycled, and a more balanced C/N ratio can reduce N removal costs. Finally, we found that the production cost of a food-grade protein-rich powder from bioethanol could compete with alternative food protein-rich products, reaching, for instance, about half of the selling price of tofu.

## MATERIALS AND METHODS

### Microorganisms.

*Methylorubrum extorquens* DSM 1338, *Wickerhamomyces anomalus* (previously known as Pichia anomala) DSM 6766, and Corynebacterium glutamicum DSM 20300 were purchased from the German Collection of Microorganisms and Cell Cultures (DSMZ, Braunschweig, Germany). *Cyberlindnera saturnus* (also known as Williopsis saturnus) CBS 254 was obtained from the CBS-KNAW Fungal Biodiversity Centre (Utrecht, Netherlands). *Metschnikowia pulcherrima* MUCL 46194 originates from the Mycothèque of the Université Catholique de Louvain (BCCM/MUCL collection, Belgium).

### Culture media.

Ammonium mineral salts (AMS) medium supplemented with vitamins was used in this study (Table SA1). To prevent substrate inhibition or toxicity, the initial concentration of ethanol (absolute ethanol, 99.5% (vol/vol); Sigma-Aldrich, Germany) was set at 4 g EtOH/L (39). The ammonium concentration was varied over the different experiments to reach a C/N molar ratio of 5, 20, and 60 (1.9; 0.47, and 0.16 g NH_4_Cl/L, respectively). C-limiting conditions were set by using a molar C/N ratio of 5 (42); dual limitation of C and N was anticipated at a molar C/N ratio of 20 (54), while N-limiting conditions were established by applying a molar C/N ratio of 60.

### Experimental procedures.

Biological triplicate experiments were conducted in 1-L Erlenmeyer flasks with a working volume of 0.30 L to determine the specific growth rate, the biomass and protein yield, as well as the nutritional charateristics of the produced MP. Prior to the cultivation in Erlenmeyer flasks, each culture was grown on LB agar (Lennox, Sigma-Aldrich, Germany) at 28°C and subsequently transferred to LB broth (Lennox, Sigma-Aldrich). Before inoculation (10% vol/vol), cultures were centrifuged at 6,603 × *g* for 5 min and washed once with 0.01 M phosphate-buffered saline (PBS, Sigma-Aldrich). The cell concentration of the inoculum was then corrected to a final optical density (OD) of 2.1 at 600 nm using a spectrophotometer (Infinite M200 Pro; Tecan, Switzerland) by resuspending the washed pellet in 0.01 M PBS. The experiments were performed by incubating the Erlenmeyer flasks at 28°C on an orbital shaker (120 rpm) with a starting pH value of 6.8. Samples (4 mL) were immediately analyzed to determine the pH and OD, after which they were centrifuged at 20,807 × *g* for 5 min and washed with 0.01 M PBS. The supernatant was collected and stored at −20°C for the determination of ethanol, acetate, and ammonium concentration. Similarly, the biomass pellet was stored at −20°C and used to identify the AA profile and quantify total carbohydrates as well as total protein. The nutritional quality assessment was performed on biomass harvested at the stationary phase.

### Analytical techniques.

The growth of microbial biomass was monitored by measuring the OD at 600 nm through a spectrophotometer (Spectronic 22; Thermo Scientific, Belgium). A calibration curve was used to convert OD to cell dry weight (CDW) for each specific microorganism. The CDW was quantified as total suspended solids (TSS) according to standard methods. A pH sensor (Orion 911600; Thermo Scientific) was used to measure pH. To determine ethanol, acetate, and ammonium concentrations, samples were first filtered (0.2-μm polyvinylidene difluoride [PVDF] filters, Chromafil). An ion chromatograph (930 Compact IC Flex; Metrohm, Switzerland) equipped with a Metrosep organic acid 250/7.8 column, a Metrosep organic acid guard column/4.6, and an ion chromatography (IC) conductivity detector (Metrohm) was used to determine the acetate concentration in each sample. The cations (Na^+^, NH4^+^) were determined using an ion chromatograph (Metrohm) equipped with a Metrosep C6-250/4.0 column and conductivity detector. Ethanol concentration was measured with a high-pressure liquid chromatography (HPLC) (Prominence-i LC-2030C Plus; Shimadzu) equipped with a refractive index (RI) detector and Rezex ROA-organic acid H^+^ (8%), 150 by 7.8 mm column, at an eluent (5 mM H_2_SO_4_) flow rate of 0.60 mL/min at 50°C. The total (crude) protein was analyzed in technical triplicates with the Pierce BCA protein assay kit (Thermo Scientific). The total carbohydrate content was determined with the method of Josefsson (55), using a spectrophotometer (Spectronic 22; Thermo Scientific) at 520 nm.

The AA profile of the cultures was determined in the stationary phase using an HPLC (Ultimate 3000; Thermo Scientific) equipped with a fluorescence detector (RF2000; Dionex) and a C_18_ column with 2.5 μm particle size (4.6 by 150 mm; XBridge, Waters), operated at an eluent flow rate of 1.85 mL/min. To measure free AA, filtered samples (0.2-μm PVDF filters; Chromafil) were derivatized with a commercial kit (AccQ-Tag, Waters) prior to injection in the HPLC. To determine the AA content of the biomass, a part of the sample was oxidized with performic acid to convert methionine to methionine sulfone and cysteine to cysteic acid and prevent the destruction of these AA during subsequent acid hydrolysis. Samples were oxidized for 16 h at 0°C followed by acid hydrolysis at 110°C for 24 h using HCl 6 M (37% fuming; ROTIPURAN, Carl Roth) under an argon atmosphere (≥99.999%, Alphagaz; Air Liquide). The essential amino acids (EAA) histidine, lysine, threonine, valine, leucine, isoleucine, and phenylalanine and the conditionally essential amino acids (CEAA) tyrosine, glycine, arginine, and proline were quantified using the same acid hydrolysis step, but without performic acid oxidation. In all cases, dl-norvaline was used as an internal standard (≥98.5%; Sigma-Aldrich).

### Cost estimation.

A cost estimation was made in order to assess the combined impact of specific growth rate, biomass yield, protein content, and nitrogen uptake efficiency for each C/N ratio. The total costs of production included operational expenses (OPEX; including nutrients, cooling water, electricity, generated electricity via anaerobic digestion, and personnel) and capital expenses (CAPEX). Costs for bioethanol, nitrogen, C removal and N removal were depicted separately (CAPEX related to the C and N removal installation were included in the total C and N removal cost and therefore excluded from the general CAPEX) (Table SA3). The utility requirements were based on material and energy balance calculations (Table SA4). The experimentally obtained data were used as starting values for these calculations. Finally, the complete mass balances of the process, based on a full-scale 150-m^3^ continuously operated bioreactor (56), were defined using Excel (Microsoft Corporation, USA) (Data Set S1). In the cases where carbon was limiting, the maximal substrate concentration in the effluent of the bioreactor was set at 0.002 kg ethanol/m^3^ to mimic the conditions of the industrial plant used by Imperial chemical industries to produce Pruteen (57). In the other scenarios, where nitrogen was limiting, the maximal concentration of total ammonium nitrogen (TAN) was set at 0.002 kg TAN/m^3^. For the initial C/N ratio of 20, the experimental results did not reveal any clear limitation from either Nor C. Here, the actual consumption of carbon and nitrogen observed from the experiments was used to identify the limiting component in the feed.

### Statistics.

Correlations between specific growth rate, biomass composition, and yields were determined using R (v3.6.1). The Spearman’s rank correlation coefficient (Spearman’s *ρ*) was determined to assess the monotonic relationship between the different variables, while the Pearson correlation coefficient (Pearson’s *r*) was used to evaluate the linearity of these variables (Table SA5). *P* values below 0.05 were considered statistically significant.

### Data availability.

The majority of the raw data are available in supplemental file 1 and 2. Other relevant data can be provided upon request.
