# Supplementary material for: The Role of Microorganisms and Carbon-to-Nitrogen Ratios for Microbial Protein Production from Bioethanol
Source: Appl Environ Microbiol. 2022 Oct 26;88(22):e01188-22. doi: 10.1128/aem.01188-22 (PMC9680612; doi:10.1128/aem.01188-22)
Supplement: Supplemental file 1 — Fig. SA1 and Tables SA1 to SA6. Download aem.01188-22-s0001.pdf, PDF file, 1.1 MB [file aem.01188-22-s0001.pdf]

Supplementary data for:

The role of microorganisms and carbon to nitrogen ratios for microbial protein production from bioethanol

---

Authors: Van Peteghem, L.,[a,b] Sakarika, M.,[a,b], Matassa S.[c], and Rabaey, K.*[a,b]

[a] Center for Microbial Ecology and Technology (CMET), Faculty of Bioscience Engineering Ghent University, Coupure Links 653, 9000 Gent, Belgium

[b] Center for Advanced Process Technology for Urban Resource recovery (CAPTURE), www.capture-resources.be, Frieda Saeysstraat 1, 9000 Gent, Belgium

[c] Department of Civil, Architectural and Environmental Engineering, University of Naples Federico II, via Claudio 21, 80125 Naples, Italy

* Correspondence to: Korneel Rabaey, Ghent University; Faculty of Bioscience Engineering; Centre for Microbial Ecology and Technology (CMET); Coupure Links 653; B-9000 Gent, Belgium; phone: +32 (0)9 264 59 76; fax: +32 (0)9 264 62 48; e-mail: Korneel.Rabaey@UGent.be; webpage: www.cmet.UGent.be.

Number of pages: 11

Number of Figures: 1

Number of Tables: 6

Calculations

Growth rate estimation

The estimation of the specific growth rate (µ) was performed as described in Candry *et al.* (2018). The OD of each sample was corrected with the control (un-inoculated condition) and subsequently log-transformed (according to commonly used population growth equations) (2). Afterwards, the Richards equation (Equation 1) was fitted to the transformed data using the nls.lm optimization algorithm from the minpack.lm package in R. From this fit, the specific growth rate for each condition could be calculated.

$$ln\left(\frac{\Delta OD}{\Delta OD_{min}}\right) = A * \left(1 + v * e^{1+v} * e^{\frac{\mu}{A} * (1+v)^{1+\frac{1}{v}} * (\lambda - t)}\right)^{-\frac{1}{v}} \quad \text{Equation 1}$$

The Richards equation estimates: carrying capacity (A), specific growth rate (µ), lag time (λ) and a shape factor with no biological meaning (v) (1).

Biomass and protein yields

Biomass yield based on C- ($Y_{X/S}$) and N-source ($Y_{X/N}$) are calculated according to equations 2 and 3. With biomass X, substrate S, and N as total ammonium nitrogen (TAN). The $CDW_i/EtOH_i/TAN_i$ and $CDW_f/EtOH_f/TAN_f$ indicate the initial (concentration at the beginning of the experiment) and final concentration (first sampling point after steady state was reached) of CDW/ethanol/TAN in the medium.

$$Y_{X/S}\left[\frac{g\ CDW}{g\ EtOH_{consumed}}\right] = \frac{\left(CDW_f[g\ TSS/L] - CDW_i[g\ TSS/L]\right)}{\left(EtOH_i[g/L] - EtOH_f[g/L]\right)} \quad \text{Equation 2}$$

$$Y_{X/N}\left[\frac{g\ CDW}{g\ TAN_{consumed}}\right] = \frac{\left(CDW_f[g\ TSS/L] - CDW_i[g\ TSS/L]\right)}{\left(TAN_i[g/L] - TAN_f[g/L]\right)} \quad \text{Equation 3}$$

Protein yield based on C- ($Y_{P/S}$) and N-source ($Y_{P/N}$) was calculated through equations 4 and 5, using the protein content P of the biomass.

$$Y_{P/S}\left[\frac{g\ protein}{g\ EtOH_{consumed}}\right] = Y_{X/S}\left[\frac{g\ CDW}{g\ EtOH_{consumed}}\right] * P\ \left[\frac{g\ protein}{g\ CDW}\right] \quad \text{Equation 4}$$

$$Y_{P/N}\left[\frac{g\ protein}{g\ TAN_{consumed}}\right] = Y_{CDW/TAN}\left[\frac{g\ CDW}{g\ TAN_{consumed}}\right] * P\ \left[\frac{g\ protein}{g\ CDW}\right] \quad \text{Equation 5}$$

Biomass quality

Given that only very few sources report the actual protein content of organisms grown on ethanol (3–5), it was decided to compare the quality of the investigated MP in terms of human nutrition with protein-rich food. As reported by the WHO/FAO/UNU (2007), an average adult (weighing 62 kg (6)) should consume about 41 g of protein on a daily basis (7). Accordingly, the amount of microbial biomass (considering 5% of moisture) necessary to cover this recommendation was calculated (7) (Figure 5A).

The nutritional quality of the biomass can be expressed via the AA score, which determines the effectiveness of meeting the essential AA at a safe level of protein intake (equation 7). A score equal to or higher than 1 indicates the AA profile fulfills or exceeds the nutritional requirement per amount of provided protein.

$$AA\ score = \frac{mg\ AA/g\ protein}{mg\ AA\ in\ required\ pattern} \quad \text{Equation 7}$$

List of Figures

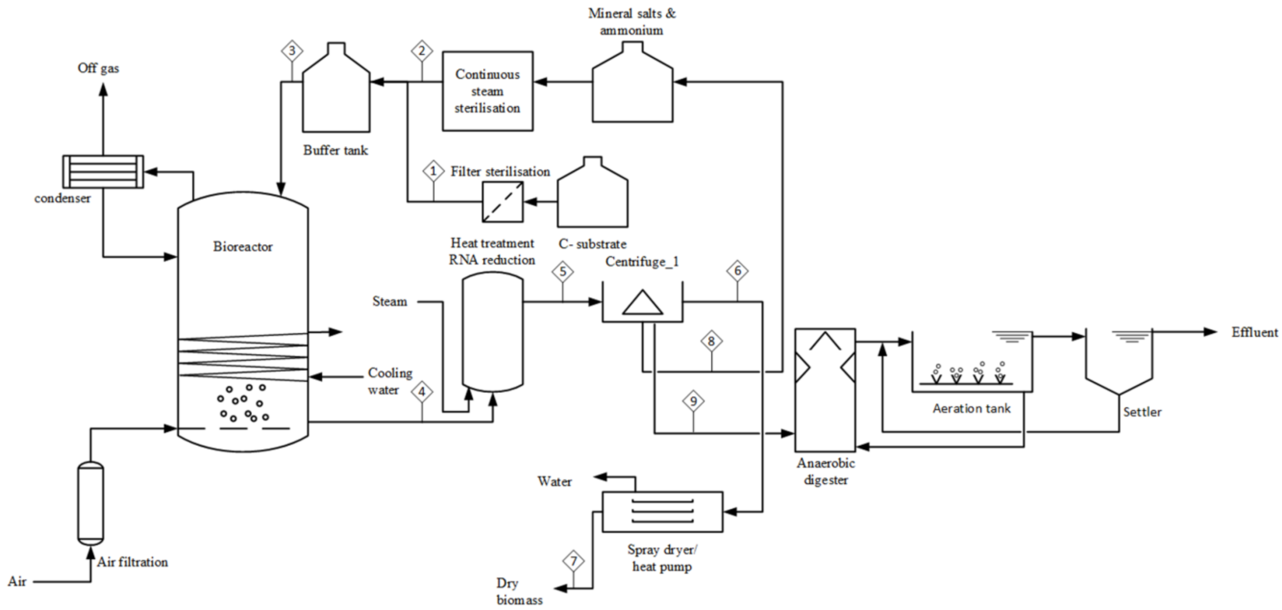

Figure A1: Generic flowsheet for the heterotrophic production process of microbial protein starting from Biotethanol. Speicific compositions and massflows for the indicated annotations are specified in supplementary data B.

List of Tables

Table A1: Final concentration of components in Ammonium mineral salts (AMS) medium and vitamins used for microbial protein production.

Compound	Unit	Value	Compound	Unit	Value
Nitrogen			Salt solution		
NH_4Cl (C/N 5)	g/L	1.9	$MgSO_4 \times 7\ H_2O$	g/L	1
NH_4Cl (C/N 20)	g/L	0.47	$CaCl_2 \times 2\ H_2O$	g/L	0.15
NH_4Cl (C/N 60)	g/L	0.16	FeNaEDTA*	mg/L	0.05
Phosphate buffer			Trace elements		
$Na_2HPO_4 \times 12\ H_2O$	g/L	2.151	Na_2EDTA* × 2 H_2O	mg/L	0.5
KH_2PO_4	g/L	0.816			
			$FeSO_4 \times 7\ H_2O$	mg/L	0.2
Vitamins			H_3BO_3	mg/L	0.03
riboflavin	mg/L	0.005	$CoCl_2 \times 6\ H_2O$	mg/L	0.02
thiamine-HCl × 2 H_2O	mg/L	0.025	$ZnSO_4 \times 7\ H_2O$	mg/L	0.01
nicotinic acid	mg/L	0.025	$MnCl_2 \times 4\ H_2O$	mg/L	0.003
pyridoxine-HCl	mg/L	0.025	$Na_2MoO_4 \times 2\ H_2O$	mg/L	0.003
Ca-pantothenate	mg/L	0.025			
biotin	µg/L	0.05	$NiCl_2 \times 6\ H_2O$	mg/L	0.002
folic acid	µg/L	0.1	$CuSO_4 \times 5\ H_2O$	mg/L	2.5
B_{12}	µg/L	0.5			

*Ethylenediaminetetraacetic acid

Table A2: Amino acid requirements for adults according to the WHO/FAO/UNU (2007) (7).

Essential amino acid	Requirements for adults (g/kg/d)	Requirements for 62 kg adults (g/day)
Histidine	10	620
Threonine	15	930
Cysteine	4	248
Tyrosine (+ phenylalanine)	25	1,550
Valine	26	1,612
Methionine	10	620
Lysine	30	1,860
Isoleucine	20	1,240
Leucine	39	2,418
Phenylalanine (+ tyrosine)	25	1,550

Table A3: Overview of units cost for all cost components.

Parameter	Value	Unit	Reference
Electricity cost	0.045	EUR/kWh$_{el}$	Energiamarkkinavirasto.
Cooling water	0.05	EUR/ton	(8, 9)
Ammonia	700	EUR/ton	(10)
Ethanol	550	EUR/ton	(11) (average of last 10 years)
Nutrients	100	EUR/ton biomass	(10)
Personnel	2	million EUR/year	(10) (Torula protein production)
N-removal	3	EUR/kg TAN	(12)
CAPEX*	70	million EUR	(10) (Torula protein production)
C-removal			
sludge treatment	280	EUR/ton	(13)
Operation	1.0	% of CAPEX	
Personnel	3.5	% of CAPEX	(14)
Maintenance	2.0	% of CAPEX	
cost UASB**	558	EUR/m^3	

*Capital expenses for MP production process excluding wastewater treatment
**Cost upflow anaerobic sludge blanket reactor

Table A4 Overview of the parameters utilized to calculate all unit operations relevant for the energy balance. The energy balance itself is used to calculate the energy requirement of the complete process.

	Energy balance components/Parameter	Value min	Value max	Unit	Notes
Filter sterilization					
	Pressure drop over membrane (ΔP)	1	2	bar	Personal communication Indusfilter.com, 2022
		101300	202600	Pa	
	Pump efficiency	0.75	0.75	%	
Steam sterilization					
	Steam requirement sterilisatoin	0.1	0.2	kg steam/ kg medium	Steam boiler producing high-pressure steam at 6 bar and feed water temperature of 20°C. Data from AB&Co, 2021
	Steam capacity	1450	160	kg/h	
	Steam compression requirement	1000	122.2	kW	
Agitation and aeration					Model used from Kreyenschulte et al., 2016
Cooling					
**T_h**	bioreactor temperature	30	30	°C	(18, 19)
**T_{ci}**	cooling water in	10	10	°C	
**COP**	coeff of performance	6	4		
**q**	specific heat of reaction	460	460	kJ/mol O_2	
**T_{co}**	cooling water out	25	25	°C	
C_{pc}	heat capacity water	4,190		kj/kg °C	
Heat treatment					
	steam requirement	0.075		kg steam/ kg culture	Values for heat treatment quorn at 74°C; 30-45 min with steam at 4 bar. (20)
	Steam capacity	1450	160	kg/h	Steam boiler producing high-pressure steam at 6 bar and feed water temperature of 20°C. Data from AB&Co, 2021.
	Steam compression requirement	1000	122.2	kW	
		0.69	1.31	kWh/ kg steam	
Centrifugation					
	Energy requirement centrifugation	0.7	2.5	kWh/m^3	Values for yeast harvesting with both nozzle centrifuge and disk-type centrifuge with 90 % effeciency. (21)
Drying					
	Energy requirement drying heat pump	0.33		kWh/kg	Low temperature drying of food, using a heat pump. Final biomass water content is set at 4 %. (22)
Anaerobic digestion					
	COD Removal eff	100		%	Assumption based on the fact that the COD is composed of unused ethanol and disintegrated biomass

Specific energy CH_4 at 35°C	38	MJ/kg CH_4	(23)
Sludge yield	0.13	kg CDW/ kg COD	
Electricity production eff	30	%	(14)
Biogas yield	0.5	m^3 Biogas/ kg COD	
CH_4 in biogas	70	%	
CH_4 volume at 35 °C	25	m^3/kmol	(23)
Molar mass CH_4	16	kg/kmol	

Table A5: Overview of performed statistical analyses and respective p-values to verify the correlation between the specific growth rate and the protein content and yield.

**Specific growth rate correlation to**	**C/N ratio**	**Pearson's correlation**			**Spearman's rank correlation**		
		t	cor	p-value	S	rho	p-value
P (g protein/ g CDW)	C/N 5	-11	-0.95	4.8E-08	974	-0.74	0.0017
	C/N 20	-2.4	-0.56	0.036	756	-0.66	0.010
	C/N 60	-0.80	-0.22	0.44	673	-0.20	0.46
Y (g protein/ g $EtOH_{consumed}$)	C/N 5	-8.6	-0.92	1.1E-06	1064	-0.90	5.7E-06
	C/N 20	1.0	0.28	0.34	252	0.45	0.11
	C/N 60	1.1	0.29	0.29	574	-0.025	0.93

Table A6: Amino acid composition and protein content of some alternative protein.

**Alternative protein**	**Unit**	**Grasshopper**	**Soft Tofu**	**Egg, raw**	**Chicken breast**
Protein content	g/g product	27	72	130	230
Serine	mg AA/ g protein	*56*	43	77	38
Glutamic acid	mg AA/ g protein	56	158	133	148
Histidine	mg AA/ g protein	56	27	25	37
glycine	mg AA/ g protein	69	36	34	44
Arginine	mg AA/ g protein	86	61	65	68
Threonine	mg AA/ g protein	106	37	44	45
Alanine	mg AA/ g protein	39	37	58	58
Proline	mg AA/ g protein	90	49	41	32
Cysteine	mg AA/ g protein	5.3	13	22	10
Tyrosine	mg AA/ g protein	76	31	40	36
Valine	mg AA/ g protein	67	46	68	52
Methionine	mg AA/ g protein	22	12	30	26
Lysine	mg AA/ g protein	63	60	72	96
Isoleucine	mg AA/ g protein	15	45	53	49
Leucine	mg AA/ g protein	70	69	87	83
Phenylalanine	mg AA/ g protein	53	44	54	40
**Reference**		(24)	(25)	(25)	(25)

References

1. Candry P, Van Daele T, Denis K, Amerlinck Y, Andersen SJ, Ganigué R, Arends JBA, Nopens I, Rabaey K. 2018. A novel high-throughput method for kinetic characterisation of anaerobic bioproduction strains, applied to Clostridium kluyveri. Sci Rep 8:1–13.

2. Begot C, Desnier I, Daudin JD, Labadie JC, Lebert A. 1996. Recommendations for calculating growth parameters by optical density measurements. J Microbiol Methods 25:225–232.

3. Yech Y. 1996. Single-cell protein of Rhodotorula sp. Y-38 from ethanol, acetic acid and acetaldehyde. Biotechnol Lett 18:411–416.

4. Mor JR, Fiechter A. 1968. Continuous cultivation of Saccharomyces cerevisiae. II. Growth on ethanol under steady-state conditions. Biotechnol Bioeng 10:159–176.

5. Abbott BJ, Laskin AI, McCoy CJ. 1973. Growth of Acinetobacter calcoaceticus on Ethanol. Appl Microbiol 25:787–792.

6. Walpole SC, Prieto-Merino D, Edwards P, Cleland J, Stevens G, Roberts I. 2012. The weight of nations: An estimation of adult human biomass. BMC Public Health 12:1–6.

7. World Health Organization, United Nations University. 2007. Protein and amino acid requirements in human nutrition.World Health Organization technical report series.

8. Pihlajaniemi V, Ellilä S, Poikkimäki S, Nappa M, Rinne M, Lantto R, Siika-aho M. 2020. Comparison of pretreatments and cost-optimization of enzymatic hydrolysis for production of single cell protein from grass silage fibre. Bioresour Technol Reports 9:100357.

9. Towler G, Sinnott R. 2021. Chemical engineering design: principles, practice and economics of plant and process design. Butterworth-Heinemann.

10. Voutilainen E, Pihlajaniemi V, Parviainen T. 2021. Economic comparison of food protein production with single-cell organisms from lignocellulose side-streams. Bioresour Technol Reports 14:100683.

11. IndexMundi. 2022. Sugar - Monthly Price - Commodity Prices - Price Charts, Data, and News. https://www.indexmundi.com/commodities/?commodity=sugar&months=120&currency=eur. Retrieved 11 January 2022.

12. Van Dongen U, Jetten MSM, Van Loosdrecht MCM. 2001. The SHARON®-Anammox® process for treatment of ammonium rich wastewater. Water Sci Technol 44:153–160.

13. European commission. 2002. Disposal and recycling routes for sewage sludge. Luxemburg.

14. Haandel A, Lubbe J. 2012. Handbook Biological waste water treatment - second edition: Design and Optimisation of Activated Sludge Systems by Van Haandel A.C. and Van der Lubbe J (2012) IWA Publishing of Alliance House, London-UK ISBN: 9781780400006 (816 pag).

15. Indusfilter.com. 2022. Personal communication.

16. AB&Co. 2021. Electric Steam Humidifiers. https://www.abco.dk/English/CustomisedBoiler/ElectricSteamBoiler/?gclid=Cj0KCQiA-aGCBhCwARIsAHDl5x_xq8Ik--U9WKzMfWJhdz7xItRaFidqIKSdZJ-ojwzctDjasaZwIucaAhI7EALw_wcB#Type_DH2. Retrieved 5 August 2021.

17. Kreyenschulte D, Emde F, Regestein L, Büchs J. 2016. Computational minimization of the specific energy demand of large-scale aerobic fermentation processes based on small-scale data. Chem Eng Sci 153:270–283.

18. Doran PM. 1995. Bioprocess engineering principles. Elsevier.

19. Jensen JK, Ommen T, Reinholdt L, Markussen WB, Elmegaard B. 2018. Heat pump COP, part 2: Generalized COP estimation of heat pump processes, p. 1136–1145. *In* 13th IIR Gustav Lorentzen Conference on Natural

Refrigerants (GL2018).

20. Ward PN. 1998. Production of food. 5,739,030. United Sates.

21. Patel MK, Crank M, Dornburg V, Hermann B, Roes L. 2004. Medium and Long-term Opportunities and Risks of the Biotechnological Production of Bulk Chemicals from Renewable Resources. Ecol Manag Restor 5:30–33.

22. Kudra T, Martynenko A. 2015. Energy Aspects in Electrohydrodynamic Drying. Dry Technol 33:1534–1540.

23. Green DW, Southard MZ. 2019. Perry’s chemical engineers’ handbook. McGraw-Hill Education.

24. Oibiokpa FI, Akanya HO, Jigam AA, Saidu AN, Egwim EC. 2018. Protein quality of four indigenous edible insect species in Nigeria. Food Sci Hum Wellness 7:175–183.

25. U.S. Department of Agriculture. 2019. FoodData Central.
